# A comparative study on acoustical properties using waste recycled porous materials for environmental sustainability

**DOI:** 10.1038/s41598-025-10065-3

**Published:** 2025-07-21

**Authors:** Vinoth Kumar Selvaraj, Jeyanthi Subramanian, Sri Sai Dutt, K. Annamalai, Elango Natarajan, S. Kumaresan

**Affiliations:** 1 School of Mechanical Engineering, Vellore Institute of Technology, Chennai, Tamilnadu India; 2 Faculty of Engineering, Technology and Built Environment, UCSI University, Kuala Lumpur, Malaysia; 3 Mechatronics, Faculty of Science and Engineering, University of Limerick, Limerick, Ireland

**Keywords:** Sustainability, Waste recycling, Biocomposites, Acoustic, RSM, COMSOL multiphysics, Engineering, Materials science

## Abstract

As noise pollution intensifies in urban areas, the need for sustainable and effective sound-reducing porous materials becomes increasingly critical. This research addresses that need by developing gypsum-based composites enhanced with vermiculite and recycled rigid polyurethane (RPU) powder, using a blend-press-sinter methodology. Gypsum-based composites were chosen for their cost-effectiveness, recyclability, structural stability, and sound-absorbing properties, all with minimal environmental impact. This approach supports the circular economy by repurposing waste materials. High-resolution scanning electron microscopy (HR-SEM) and Fourier transform infrared spectroscopy (FTIR) were employed to analyze the size, structure, uniformity, and presence of organic and inorganic compounds. Using response surface methodology (RSM) for optimization, the ideal formulation for the noise reduction coefficient (NRC) was identified, with an optimal mix of 5.3 wt% vermiculite and 6.5 wt% RPU, achieving an NRC value of 0.3628. Acoustic simulations using COMSOL Multiphysics, guided by the Johnson-Allard model, demonstrated that the optimized composite effectively reduced sound pressure levels by 17 to 58 dB across the 200 to 2000 Hz frequency range. These findings underscore the composite’s potential for room acoustics applications. By incorporating recycled and natural materials, this approach not only enhances acoustic performance but also promotes sustainable material practices.

## Introduction

 In recent years, many studies have been carried out to improve acoustic insulation, with the focus on sustainable porous materials. Acoustic insulation is very important in enhancing sound quality and reducing noise pollution in various places. Researchers are increasingly interested in gypsum composites for improving sound absorption and insulation, and for contributing greener environment by applying natural and secondary materials in building processes. Noise pollution in modern times has emerged as a significant problem since cities continue to expand and technologies develop with great speed^1^. Excessive noise may raise health risks such as loss of hearing, stress, and even destruction of industrial buildings^2,3^. Being acutely aware of the problem, governments have started to pass strict noise regulations, which has caused great interest in searching for effective, sustainable materials to reduce noise^4^.

Synthetic acoustic absorbers, such as polyurethane foams and polyester fibers, have been widely utilized in noise control applications due to their lightweight and cost-effective properties. However, these materials often exhibit limitations in sound absorption performance, particularly at specific frequency ranges, and raise environmental concerns due to their non-biodegradable nature. Consequently, there is a growing interest in exploring sustainable alternatives that offer enhanced acoustic properties while being environmentally friendly. Several studies have evaluated the sound absorption performance of synthetic materials. For instance, Liang et al. (2022) reviewed various synthetic fiber materials and noted that, despite structural modifications, these materials often suffer from low sound absorption coefficients (SAC) and narrow effective frequency bands, limiting their industrial noise reduction capabilities^5^. Similarly polyurethane foams, while commonly used, exhibit moderate acoustic performance, with SAC values typically ranging between 0.4 and 0.6 at mid to high frequencies^6^. In contrast, natural fiber-based composites, such as those made from coir or jute, have demonstrated superior sound absorption properties. For example, Muralidharan (2024) reported that coir-based materials achieved SAC values exceeding 0.9 at certain frequencies, outperforming many synthetic counterparts^7^.

Gypsum is important for its usage in various industries focused on acoustical performance due to its abundance, low cost, and absorption ability^8^. In construction, it is a core material in drywall systems and plasters that reduce noise pollution through sound absorption, particularly in residential homes, commercial buildings, and institutions^9^. Acoustic qualities of this material find application in recording studios, theatres, and concert halls through damping echoes and improving sound^10^. Gypsum-based barriers are used in industrial plants to reduce the noise generated by machinery, thus ensuring safety and compliance^11^. Such gypsum products also find applications in transport infrastructure, in schools, health institutions, and workplaces to reduce noise to ensure comfort and productivity^12,13^. Recently, vermiculite and recycled rigid polyurethane (PU) powder have been studied as additives in improving the sound absorption efficiency of the composites. Vermiculite is a lightweight porous mineral of thermal stability, which possesses some good sound-absorbing qualities^14,15^. This mineral has found applications in the construction, automotive interior, and appliance insulation industries for the reduction of noise and improvement of thermal comfort^16,17^. In agriculture, vermiculite is used for soil conditioning and the noise absorption of greenhouses^18^. It also provides good fireproofing, which is suitable for use in safes, furnaces, and fire doors^19,20^. The effective material attributes in the vermiculite category are said to create value for the material to improve any service in thermal and acoustical performance^21^.

Recycled Rigid Polyurethane (RPU) powder, which comes from post-consumer polyurethane foam scrap, serves as an environmentally friendly replacement for a filler or additive, replacing an assortment of applications. Because recycling of PU foams can be achieved by three techniques, namely, mechanical, chemical, and thermal techniques, each unique method has its advantages and disadvantages^22^. The mechanical method includes crushing and grinding foams into numerous fragments, turning them into powder, thus usable for insulation materials and carpet underlay^23^. It is cost-effective, energy-efficient, and easy to carry out, although the recycled material may be of low quality because of contamination^24^. Chemical recycling in general breaks the PU foam down to its elemental components, such as polyols and isocyanates, and can be reused in the process of the production of new polyurethane foams^25^. It is possibly more suitable for ensuring the highest quality of recycling, although very complex equipment is needed, which could be expensive to carry out, and might cause environmental and safety concerns^26^. Thermal recycling, otherwise known as pyrolysis, is a process in which the PU foams are treated at high temperatures in the absence of oxygen until they break down to gaseous, liquid, and solid products, which are then used as feedstock materials for new materials or for energy recovery^27,28^. While this method is efficient and can handle a variety of foam types and contaminants, it is energy-intensive and may generate emissions requiring treatment^29^. The most suitable method of recycling will depend on the requirements for the final product in terms of quality, the resources available, and the impact on the environment^30^. Recycled RPU powder is ideal for acoustical applications, offering enhanced mechanical properties, such as strength and durability, while effectively absorbing sound waves to reduce noise and reverberation^31^. It’s thermally stable and ensures consistent acoustical performance across temperature changes, while its smooth surface allows for versatile aesthetic finishes^32^. RPU powder presents an exciting opportunity for creating quiet, comfortable, and visually appealing acoustic solutions in spaces like recording studios, theatres, and offices^33^. Gypsum-based materials have been shown to exhibit high porosity, achieving sound absorption coefficients over 90% for frequencies below 1000 Hz when perforated^34,35^. Vermiculite has also proven effective in sound absorption, particularly when used as a filler in bio-based composites^36^. Studies have shown that vermiculite enhances mechanical stability, sound absorption, and microbiological resistance, contributing to sustainability by acting as carbon storage^37^. In combinations like vermiculite and coconut coir fibres, the material has shown an 18.7% improvement in acoustic absorption, making it a competitive alternative to conventional sound insulation options^38^. Studies combining RPU with fabrics and bamboo fibres have shown improved sound absorption, particularly at mid and low frequencies^39^. Additionally, rebonded RPU foams have achieved SAC above 0.8 over a wide frequency range, highlighting their adaptability and effectiveness in noise control. To optimize the NRC of gypsum composites, we utilized a statistical technique called RSM with a CCD, a type of design of experiments (DOE) dealing with the process of investigation and optimization of relationships between multiple input variables and their effects on one or more output responses^40^. RSM explores these interactions systematically, often by use of polynomial designs, so that optimal conditions are identified by a minimum number of experiments^41^. This technique is therefore very useful, especially during iterative refinement of an experimental setup^42^. It is also very useful in cases involving complex systems and when resources are constrained, where it becomes very important to maximize a response using as few trials as possible^43^.

The Johnson-Allard model is a tool originally developed to simulate acoustic propagation in porous media, including foams and fibres^44^. This model relies on a porous and rigid material framework, where the material’s rigid frame supports a network of interconnected pores. These pores allow air and sound waves to penetrate the material, interact with the solid framework, and contribute to the overall acoustic behaviour. The most important parameters for implementing this kind of model are flow resistivity, surface tortuosity, porosity, thermal and viscous characteristic lengths^45^. Porosity is the ratio of the pore volume to the total volume. It directly affects sound absorption^46^. Flow resistivity refers to the measurement of how much a material resists the passage of airflow through it. The higher the flow resistivity, the higher the sound absorption^47^. Tortuosity is a measure of how tortuous the average path of airflow is through the pore space of the material^48^. Generally, the more convoluted the path, the longer it takes to flow through the material, which results in more interactions between the sound wave and the material. These interactions cause the sound energy to be dissipated, which results in absorption. Viscous and Thermal Characteristic Lengths are lengths associated with the friction and heat transfer effect, which contain the energy dissipation mechanisms^49,50^. However, an inverse acoustic characterization method has been developed to estimate these parameters from experimental data by minimizing the difference between model predictions and actual measurements. COMSOL Multiphysics is a software application utilized for FEM analysis and multi-physics simulations, supporting a wide variety of applications in mechanics, fluid dynamics, and acoustics^51,52^. Praised by many for its very user-friendly interface and ease of coupling of multiple physical phenomena in one model, COMSOL has become a favourite tool in academic research and education^53^. Though powerful and industry-focused, Ansys ranks below COMSOL in terms of popularity within the academic community, precisely because of its relative user-friendliness and flexibility, making COMSOL well-suited to solve several complex cross-disciplinary problems^54,55^.

The novelty of this research lies in its response to the growing need for sustainable soundproofing solutions to combat the rising levels of noise pollution in urban environments. By developing gypsum-based composites enhanced with vermiculite and recycled rigid polyurethane (RPU) powder, this study proposes a blend-press-sinter methodology that optimizes the cost-effectiveness, recyclability, and sound-absorbing properties of gypsum while ensuring structural stability. The use of recycled materials, such as RPU powder, aligns with the principles of a circular economy, reducing waste and promoting sustainability. To maximize the acoustic performance, the study employs Response Surface Methodology (RSM) to identify the optimal Noise Reduction Coefficient (NRC) value. Additionally, real-time simulations of room acoustics are conducted using COMSOL Multiphysics, providing valuable insights into the performance of these composites in various environmental settings^56,57^. This innovative approach not only addresses environmental concerns by reusing waste materials but also contributes to the development of effective, eco-friendly sound insulation solutions for a wide range of applications.

## Methodology

### Materials

Gypsum was procured from Kaoni and Randhisar Gypsum Mines, India with an average size of 6.2 ± 1.5 μm. Commercially available Vermiculite with an average size of 7.3 ± 1.2 μm is procured from Sri Ramamurthy Vermiculite Mines, India. The waste rigid polyurethane was acquired from ASL DRDO, Hyderabad, and processed through recycling with an average size of 254 ± 18 μm. Their properties are mentioned in Table 1.

**Table 1:** Fillers and their properties.

Fillers	Particle size (µm)	Density (g/cm^3^)	Curing Temperature (°C)
Gypsum	6.2 ± 1.5	2.3	150
Vermiculite	7.3 ± 1.2	2.4–2.7	150
RPU powder	254 ± 18	1.08	150

### Preparation of gypsum composites

**Fig. 1:**
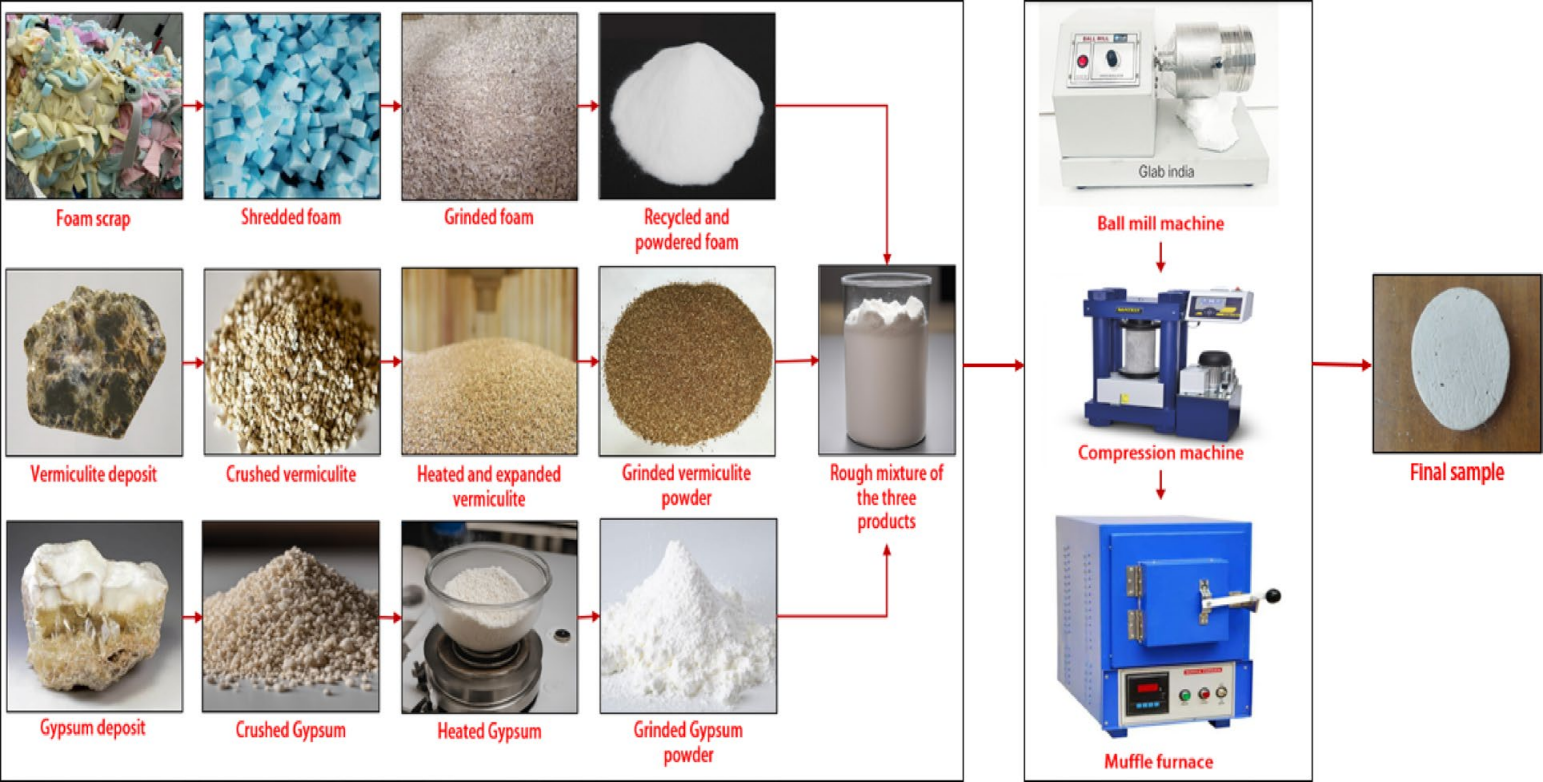
Flow chart for the gypsum composite production process.

**Fig. 2:**
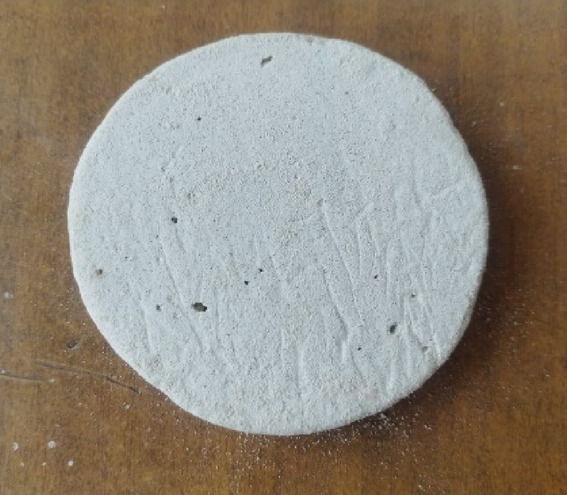
Final sample of gypsum composites.

The manufacturing process of gypsum composites starts with selecting and procuring the major raw materials used: gypsum, vermiculite, and recycled rigid PU powder. The first process in manufacturing composite is grinding and mixing of ingredients to get a uniform distribution of each ingredient. The RPU was grinded using an ADIT grinder, and the vermiculate particles were grinded using a pulverizer and turned into smaller particles. The gypsum, vermiculite, and RPU particles were sieved using a RETSCH AS 200 basic vibratory sieve shaker for 20–30 min. The gypsum and vermiculate obtained are around 1–10 μm, and the RPU powder processed is around 200–300 μm. After sieving, powder mixing was performed using a Fritsch Pulverisette 6 classic line planetary ball mill with a powder-to-ball ratio of 10:1 made of titanium balls, operating at 300 rpm for 60 min, ensuring uniform distribution of fillers in the gypsum and consistent composite properties. After grinding and mixing, the mixture is poured into silicon moulds, provided by Chemzest Techno Products PVT LTD, India. These silicone molds are created by pouring silicone into 3D-printed models with the exact dimensions, allowing them to cure until hardened. Once cured, the silicone molds are removed and used for fabricating the samples. After that, it is compressed using a Shimadzu Universal Testing Machine (Model: AG-X Plus, 50 kN capacity) at a pressure of 10 MPa, which will allow us to achieve the desired measurements of 100 mm across and 20 mm thick., to maintain the material’s acoustic properties by preserving some air pockets within the structure, which can be beneficial for sound absorption. This ensures high density in the composite and gives strength to the final product. The green compact sample is then taken out and cured in a Nabertherm LHT 02/17 LB muffle furnace at 150 ℃ for 2 h to improve the strength and stability of the composite. At this point, any remaining moisture is driven off, and the material achieves its final properties. Figure 1 illustrates the flow chart for the gypsum composite production process. Figure 2 shows an image of the final sample of gypsum composites.

### Acoustic setup

**Fig. 3:**
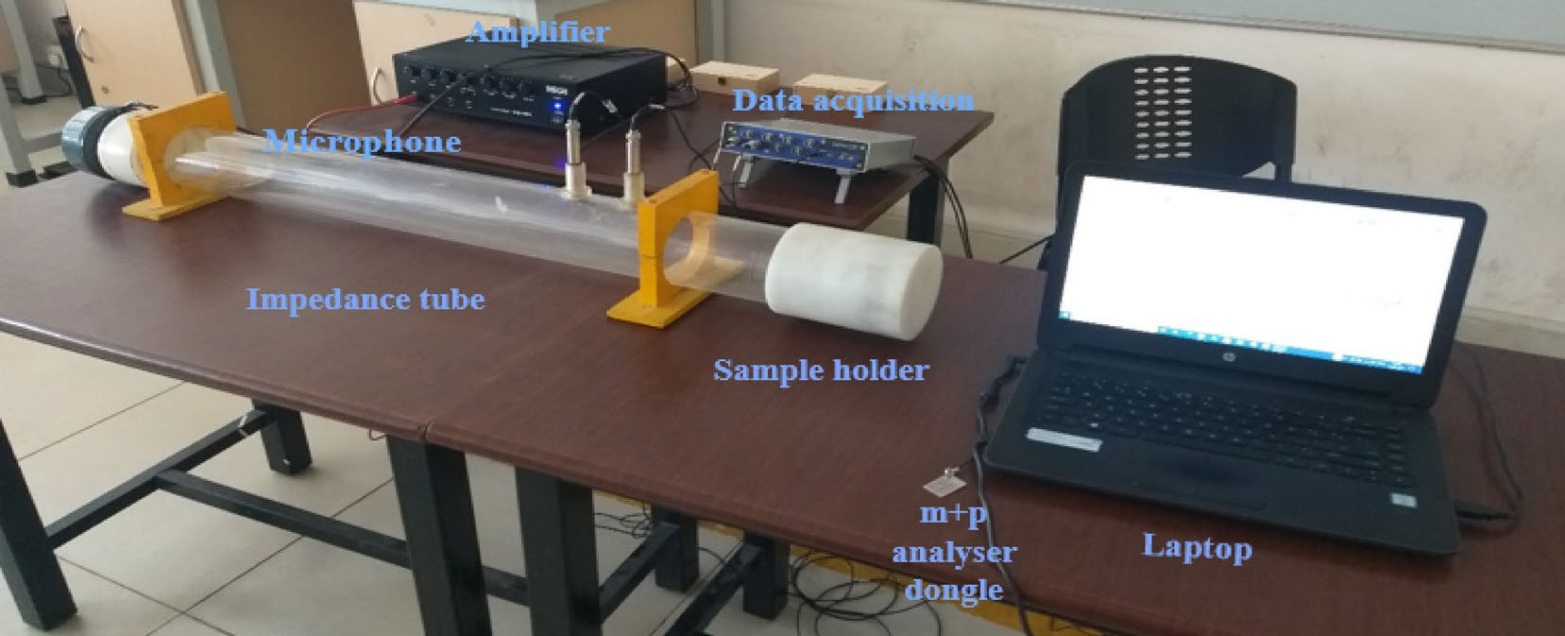
Impedance tube test setup.

This study focuses on evaluating the sound absorption properties of the developed composite using a custom-built impedance tube setup following ASTM E1050-12 standards. Sound absorption—defined as the reduction in sound power levels within an environment—differs from sound insulation, which blocks sound transmission between spaces. The impedance tube used in this study is made of acrylate for optimal acoustic performance and durability, with an internal diameter of 100 mm and a length of 900 mm. A 16 Ω speaker capable of generating sound between 200 Hz and 2000 Hz is mounted on the left end, and a cylindrical test sample of 100 mm diameter is placed at the right end. Two Microtech Gefell microphones are positioned at the center of the tube and are connected to an M + P Vibpilot data acquisition system. The entire setup is controlled via a computer running MATLAB, which facilitates automated signal acquisition and processing. Time-domain signals are captured using M + P spectrum analyzer software with a frequency resolution of 0.5 Hz. The Sound Absorption Coefficient (SAC) is computed in MATLAB using transfer function equations derived from the microphone data. Each test is repeated to ensure accuracy, and average SAC values are reported. The complete test configuration is shown in Fig. 3.

According to the ASTM E1050 and ISO 10534-2 standards, the usable frequency range of an impedance tube is determined by its diameter. For a 100 mm diameter tube, the reliable frequency range falls between 200 Hz and 2000 Hz. This range ensures that only plane waves propagate within the tube and avoids interference from higher-order acoustic modes. The cutoff frequency for the first non-planar (radial) mode is given by the following formula:

1 $$\:{f}_{c}=\:\frac{1.841*c}{\pi\:*D}$$

Where $\:{f}_{c}\:$ is the cutoff frequency (Hz), $\:c$ is the speed of sound in air (typically ~ 343 m/s), and $\:D$ is the internal diameter of the tube (in meters).

For a tube diameter of 100 mm (0.1 m), this yields a cutoff frequency of approximately 2008 Hz. Frequencies below ~ 200 Hz are limited by the length of the sample and the signal-to-noise ratio^58^. Table 2 below summarizes the practical frequency limits for different tube diameters:

**Table 2:** Practical frequency ranges for impedance tubes of different diameters.

Tube diameter (mm)	Lower frequency Limit (Hz)	Upper frequency limit (Hz)
29	~ 500	~ 6400
50	~ 250	~ 4000
100	~ 200	~ 2000

The transfer function was estimated from the data obtained from the two microphones, following Eqs. 2 and 3.

2 $$\:{k}_{0}=\frac{2\pi\:f}{{c}_{0}}$$

where $\:{k}_{0}$​ is the wave number, *f* is the frequency, and $\:{c}_{0}$​ is the speed of sound.

3 $$\:{H}_{12}=\left(\frac{p2}{p1}\right)=\frac{{e}^{j{k}_{0}{x}_{2}}+r{e}^{-j{k}_{0}{x}_{2}}}{{e}^{j{k}_{0}{x}_{2}}+r{e}^{-j{k}_{0}{x}_{1}}}$$

where, $\:{H}_{12}$ is the acoustic transfer function, $\:p2$ and $\:p1$ are the acoustic pressures of two microphones, $\:{x}_{1}$ and $\:{x}_{2}\:$are the distances between the location of the sample *x* = 0 and the two microphones. The reflection and absorption coefficients are calculated using Eqs. 4 and 5.

4 $$\:R=\:\left(\frac{{e}^{-jkS}-\:H12}{H12-{e}^{jks}}\right)*{e}^{2jk\left(l+S\right)}$$

where, $\:R$ is the Reflection coefficient.

5 $$\:\alpha\:=1-\mid\:R{\mid\:}^{2}$$

Where, is the Absorption coefficient.

The Sound Absorption Coefficient (SAC) can be determined using Eq. 5, where *R* represents the complex sound reflection coefficient, derived from Eq. 4. This coefficient is the ratio of reflected to incident pressure.

6 $${\text{NRC}} = \:\frac{{\alpha \:_{{250}} + \alpha \:_{{500}} + \alpha \:_{{1000}} + \alpha \:_{{2000}} }}{4}$$

where $\:{\alpha\:}_{250}$​, $\:{\alpha\:}_{500}$​, $\:{\alpha\:}_{1000}$​, and $\:{\alpha\:}_{2000}$​ are the Sound Absorption Coefficients (SAC) at 250, 500, 1000, and 2000 Hz frequencies respectively.

To assess the accuracy of the experimental results, the percentage error between the actual and predicted values of the NRC was calculated using the equation:

7 $$Error\;(\% ) = (NRC_{{actual}} - NRC_{{{\text{predicted}}}} )\backslash NRC_{{actual}}$$

Where $\:{NRC}_{actual}$ and $\:{NRC}_{predicted}\:$are Actual and Predicted value of NRC obtained from the experimental statistical analysis.

Equation 6 provides the method to obtain the NRC, whereas Eq. 7 determines the percentage of error between the actual and predicted NRC^59^.

### Response surface methodology (RSM)

Response Surface Methodology (RSM) was used to optimize the formulation of gypsum-based composites by evaluating the effects of vermiculite and recycled RPU powder content on the Noise Reduction Coefficient (NRC). A Central Composite Design (CCD) was employed to develop a second-order polynomial model for predicting NRC values. The experimental design, based on the Design of Experiments (DOE) methodology, considered input parameters such as the weight percentages of vermiculite, RPU powder, and gypsum, with NRC as the output across 13 samples. The design and analysis were conducted using Minitab software, which also calculated the density of each sample. Table 3 presents the composition of the samples, where S0 is the control (pure gypsum), and S1–S13 represent different proportions of vermiculite and RPU powder. These variations lead to differences in material interactions, phase distribution, and microstructure, affecting properties like density and acoustic absorption. The regression equation (Eq. 8) models NRC based on the weight percentages of vermiculite and RPU powder. The quadratic model was statistically significant for all parameters, confirming that the chosen filler proportions provided effective acoustic shielding^60^.

8 $$\begin{aligned} \:ActualNRC & = & & & \\ & + 0.4897\: - \:0.05420\:Vermiculite\:\left( {wt.\% } \right) \\ & + \:0.00302\:Rigid\:PU\:Powder\:\left( {wt.\% } \right) \\ & + \:0.001732\:Vermiculite\:\left( {wt.\% } \right)*Vermiculite\:\left( {wt.\% } \right) \\ & - \:0.000182\:Rigid\:PU\:Powder\:\left( {wt.\% } \right)*Rigid\:PU\:Powder\:\left( {wt.\% } \right) \\ & + \:0.000560\:Vermiculite\:\left( {wt.\% } \right)*Rigid\:PU\:Powder\:\left( {wt.\% } \right) \\ \end{aligned}$$

**Table 3:** Density measurement for L_13_ orthogonal array.

Specimen	Vermiculite (Wt.%)	Rigid PU powder (Wt.%)	Gypsum (Wt.%)	Density (kg/m^3^)
S0	–	–	100	1520
S1	5	10	85	1532
S2	15	10	75	1317
S3	5	30	65	1306
S4	15	30	55	1710
S5	5	20	75	1542
S6	15	20	65	1173
S7	10	10	80	1319
S8	10	30	60	1216
S9	10	20	70	1172
S10	10	20	70	1500
S11	10	20	70	1516
S12	10	20	70	1483
S13	10	20	70	1422

### Inverse characterization using GA

In this study, the design parameters for acoustic optimization were determined using a genetic algorithm (GA), which effectively addressed the complexity and constraints associated with inverse characterization problems^61,62^. The non-acoustic properties of the gypsum-based composite—flow resistivity (σ), thermal characteristic length (Λ′), viscous characteristic length (Λ), porosity (ϕ), and tortuosity (α∞)—were estimated using a GA-based MATLAB program incorporating the Johnson-Champoux-Allard (JCA) model. These parameters are critical for accurate acoustic simulation in COMSOL Multiphysics. The inverse characterization employed standard input parameters, including air density (ρ_air = 1.21 kg/m³), dynamic viscosity (η = 1.84 × 10⁻⁵ Ns/m²), Prandtl number (Pr = 0.702), specific heat ratio (γ = 1.4), and atmospheric pressure (P₀ = 101,320 N/m²)^63^. The GA facilitated robust convergence, enabling precise modeling of acoustic behavior in porous gypsum composites^64^. The parameters are very important in simulating the acoustic behavior of the gypsum composite in COMSOL Multiphysics.

9 $$\:{F}_{m}=\sum\:n\mid\:{\alpha\:}_{exp}\left({\omega\:}_{n}\right)-{\alpha\:}_{mod}\left({\omega\:}_{n}\right)\mid\:$$

Where $\:{F}_{m}$ is the cost function that represents the total error or difference, $\:\sum\:n$ is the summation of all data points n, $\:{\alpha\:}_{exp}\left({\omega\:}_{n}\right)$ is The experimental value of the property α at angular frequency $\:{\omega\:}_{n}$, $\:{\alpha\:}_{mod}\left({\omega\:}_{n}\right)$ is the model-predicted value of the property α at an angular frequency $\:{\omega\:}_{n}$.

10 $$\:{p}_{eff}\:=\:\:\frac{{\alpha\:}_{\infty\:}{p}_{air}}{{\upvarphi\:}}\left[1+\:\frac{\sigma\:\varphi\:}{j\omega\:{\alpha\:}_{\infty\:}{p}_{air}}\sqrt{1+\frac{j4{{\alpha\:}_{\infty\:}}^{2}{\upeta\:}{p}_{air\omega\:}}{{\sigma\:}^{2}{{\Lambda\:}}^{2}{{\upvarphi\:}}^{2}}}\right]$$

Where $\:{p}_{eff}$ is the Effective density of the porous material, $\:{\alpha\:}_{\infty\:}$ is Tortuosity, $\:{p}_{air}$ is the density of air (1.21 kg/m³), $\:{\upvarphi\:}$ is Porosity, $\:\sigma\:$ is Flow resistivity, $\:j\:$ is an imaginary unit used to denote complex numbers, $\:\omega\:$ is the angular frequency, $\:{\upeta\:}$ is the viscosity of air (1.84 × $\:{10}^{2}$) Ns/m²), $\:{\Lambda\:}\:\text{i}\text{s}\:\text{V}\text{i}\text{s}\text{c}\text{o}\text{u}\text{s}\:\text{c}\text{h}\text{a}\text{r}\text{a}\text{c}\text{t}\text{e}\text{r}\text{i}\text{s}\text{t}\text{i}\text{c}\:\text{l}\text{e}\text{n}\text{g}\text{t}\text{h}.$

11 $$\:{K}_{eff}\:=\:\frac{\gamma\:{p}_{0}/\varphi\:}{\gamma\:(\gamma\:-1)[1+\:\text{}\text{}\frac{\sigma\:\varphi\:}{j\omega\:{\alpha\:}_{\infty\:}{p}_{air}}\sqrt{1+\frac{j4{{\alpha\:}_{\infty\:}}^{2}{\upeta\:}{p}_{air\omega\:{p}_{r}}}{{\sigma\:}^{2}{{\Lambda\:}}^{2}{{\upvarphi\:}}^{2}}}]\:}$$

Where, $\:{K}_{eff}$ is the effective bulk modulus of the porous material, $\:\gamma\:$ is the specific heat ratio of air (1.4), $\:{p}_{0}$ is the atmospheric pressure, $\:{p}_{r}$ is atmospheric pressure (101,320 N/m²).

12 $${\text{Z}} =\:\sqrt{{p}_{eff}*\:{K}_{eff}\:}$$

Where Z is the Acoustic impedance.

13 $${\text{K}} = \:\omega\:\sqrt{\frac{{p}_{eff}}{{K}_{eff}}}$$

Where K is the Wave number.

### Computational simulation

COMSOL Multiphysics was used to simulate and optimize the acoustic properties of gypsum-based composites and recycled materials, focusing on sound propagation, absorption, and reflection. The finite element model adhered to the six-elements-per-wavelength rule to ensure reliable results within the 200–2000 Hz frequency range. Variations in material composition, density, and thickness of gypsum-based composites were analyzed for their impact on sound absorption. The acoustic properties of recycled materials were compared to gypsum composites to assess their potential for sound insulation applications^65^. Parametric analysis and design optimizations were conducted to refine material composition and geometry for enhanced sound absorption efficiency. This research highlights the feasibility of using recycled construction and demolition waste as a sustainable alternative to conventional sound-insulating materials.

## Results and discussion

### High-resolution scanning electron microscope (HR-SEM)

**Fig. 4:**
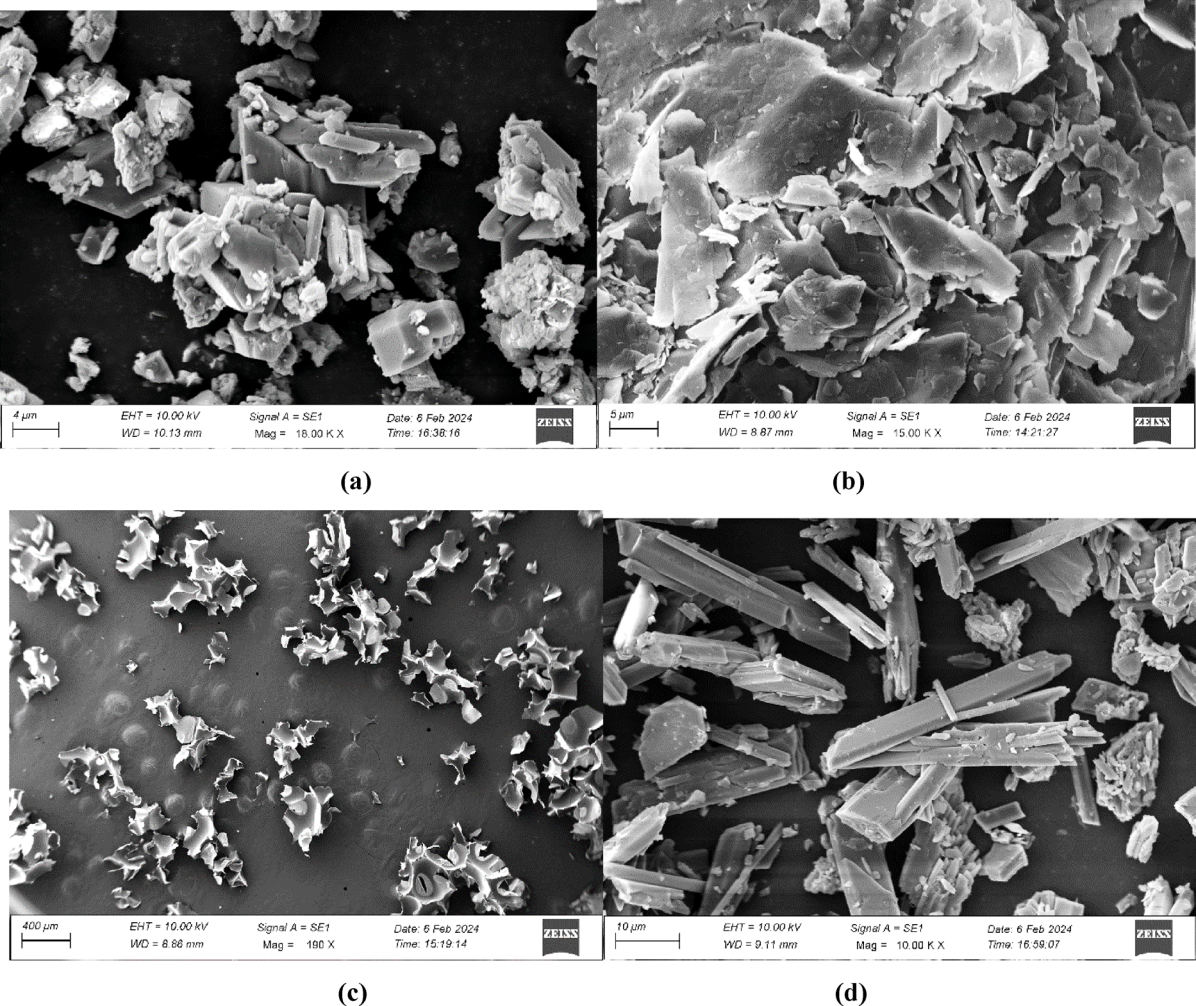
HR-SEM characterization of (**a**) Gypsum, (**b**) Vermiculate, (**c**) RPU Powder, **b**) Gypsum with fillers.

High-resolution Scanning Electron Microscopy (HR-SEM) analysis was conducted to study the microstructure, dispersion of fillers, and pore morphology of the gypsum composite samples. The specimens were first sectioned to appropriate dimensions (~ 10 mm × 10 mm) and air-dried for 24 h to remove any residual moisture. To ensure electrical conductivity and high-resolution imaging, the samples were mounted on aluminum stubs using carbon adhesive tape and subsequently sputter-coated with a ~ 10 nm layer of gold using a Quorum Q150R ES sputter coater. SEM imaging was performed using a ZEISS EVO 10 scanning electron microscope equipped with a LaB₆ electron source, operating in secondary electron (SE) mode. The EHT was set at 10 kV, with a working distance of 8–11 mm, and the chamber was maintained at a high vacuum (~ 10⁻⁵ mbar). Images were captured at varying magnifications ranging from 150× to 18,000×, depending on the structural features being analyzed. Image acquisition was carried out using the integrated SmartSEM software, ensuring consistent contrast and brightness across all samples. These images provide qualitative insights into the homogeneity of the filler dispersion and the porous architecture crucial to acoustic performance. Gypsum particles exhibit a granular or blocky structure with smooth surfaces, varying in shape and size^66^. Pores and cavities in gypsum also differ in size and have developed thickness. Depending on the place of origin of the gypsum and the method of its processing, the size and thickness of pores differ. Pores can have a size from 1 to 10 micrometers and developed thickness^67^. Upon close observation, the presence of Gypsum can be confirmed by inspecting Fig. 4a. Vermiculite shows characteristic flaky and layered morphology^68^. The individual layers are very thin and can range from a few micrometers up to several millimeters, but typically fall within the range of 1 to 10 micrometers for smaller particles. Pores and voids within the Vermiculite can be in micron sizes^69^. This can be observed, and its presence can be confirmed by looking at Fig. 4b. The particles of RPU powder have an uneven, granular shape with rough surfaces. The individual particles are irregular in shape and different from each other. The average lateral dimensions of the individual particles usually lie in the range between 200 and 500 micrometers. Pores and voids may be present within the particles of the RPU powder^70^. Pores can be tiny – ranging a few micrometers in diameter, and of course, thicknesses change accordingly. The changes in pore characteristics are directly influenced by factors such as the type of recycling processes used, and the material of the polyurethane used. These characteristics, in turn, influence the pore size and thickness^71^. This can be observed, and the presence of RPU powder is confirmed by looking at Fig. 4c. Figure 4d presents the HR-SEM image of gypsum combined with Vermiculite and RPU powder fillers. The image shows that the fillers are evenly spread and uniformly distributed throughout the gypsum matrix. Vermiculite flakes and RPU powder particles are well-integrated, indicating a homogeneous composite.

### Fouriertransform infrared spectroscopy (FTIR) characterization

**Fig. 5:**
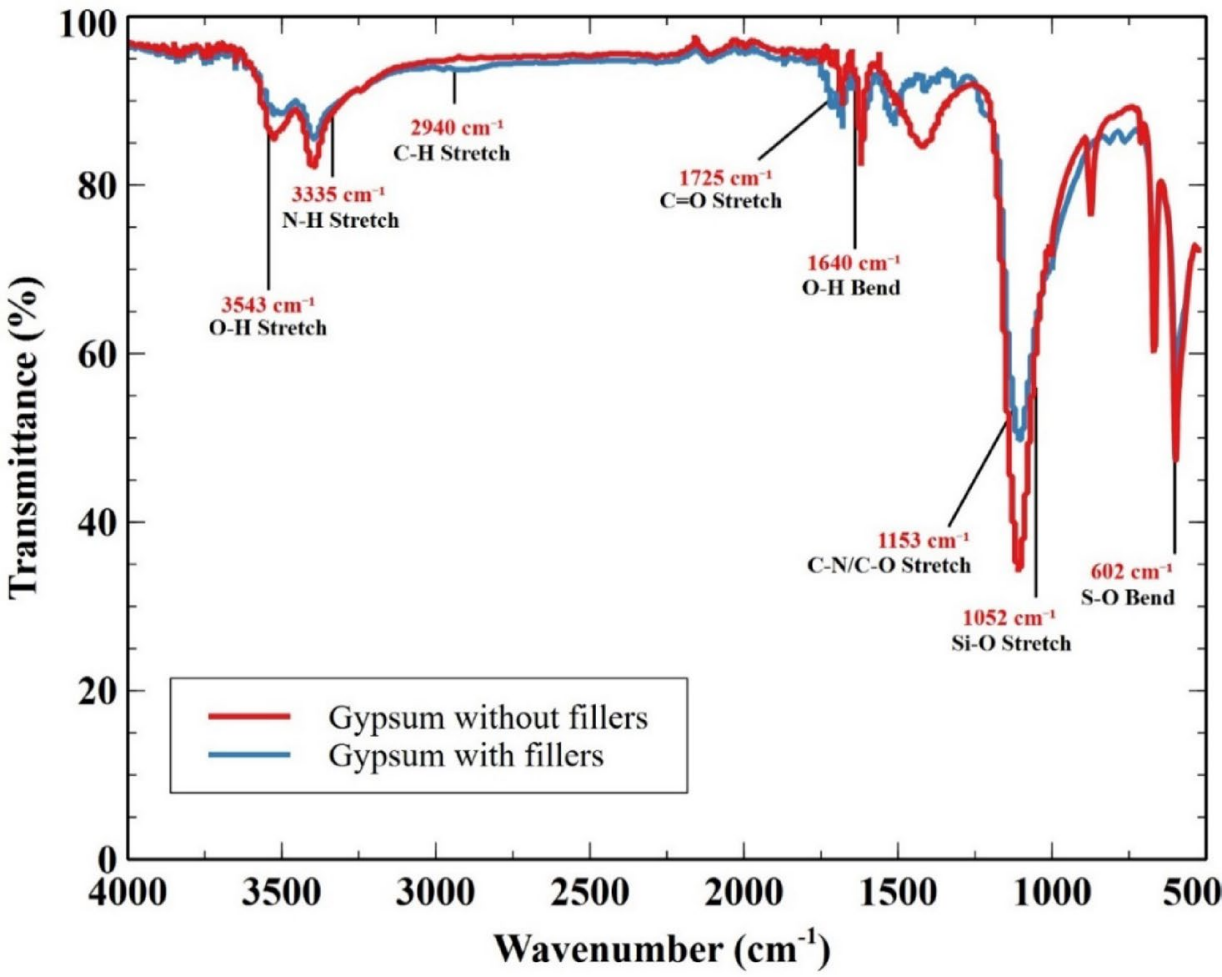
FTIR analysis.

FTIR analysis was performed using a Shimadzu IR Affinity-1 spectrometer, operating in the mid-infrared range (4000–400 cm⁻¹), to examine the chemical interactions and structural characteristics of gypsum composites with and without fillers. This technique helps assess the retention or modification of chemical bonding due to the incorporation of recycled polyurethane (RPU) powder and vermiculite, ensuring material stability and performance. The spectrum reveals significant absorption bands corresponding to key functional groups, highlighting the chemical contributions of gypsum, RPU powder, and vermiculite. In the gypsum without fillers, the key peaks observed include the O-H stretching vibrations at 3543 cm⁻¹, which are indicative of water molecules within the gypsum structure. The S-O bending mode at 602 cm⁻¹ is characteristic of the sulfate group, confirming the presence of gypsum. Upon adding fillers, the spectrum shows additional peaks corresponding to the functional groups present in RPU powder and vermiculite. The N-H stretching vibration at 3335 cm⁻¹ is a prominent feature attributed to the urethane and urea groups in the RPU powder. Similarly, the C-H stretching vibration at 2940 cm⁻¹ is also associated with the alkane groups in RPU powder. The introduction of vermiculite is evidenced by the O-H bending vibration at 1640 cm⁻¹, which corresponds to water molecules within the vermiculite structure. Additionally, the C = O stretching vibration at 1725 cm⁻¹, related to the ester bond in RPU powder, and the Si-O stretching vibration at 1052 cm⁻¹, likely from the silicate layers in vermiculite, further confirm the presence of these fillers. The combination of these materials with gypsum results in a composite with a more complex spectral profile, indicating successful integration and the formation of new interactions between the gypsum matrix and the fillers. This analysis highlights the enhanced functionality and potential application of gypsum-based composites with RPU powder and vermiculite for improved acoustic insulation and sustainability. The above conclusions can be drawn from Fig. 5^72^.

### Acoustical experimental analysis

Acoustic experimental test analysis was carried out based on ASTM E 1050-12 standard. The testing setup consists of MATLAB software, two microphones, a data acquisition (DAQ) system, a computer, a speaker, and an impedance tube, all used to analyze acoustic characteristics across frequencies from 200 to 2000 Hz. A 16 Ω speaker was installed at one end of the impedance tube to generate sound waves. Two half-inch Microtech Gefell microphones were positioned inside the tube and linked to the M + P Vibpilot DAQ system. The samples were shaped into spheres with a diameter of 100 mm and placed at the opposite end of the tube. Every test was conducted multiple times to ensure accuracy, and the average values were recorded. MATLAB software was employed to compute the Sound Absorption Coefficient using the provided equations. The signals recorded in the time domain were analyzed using M + P spectrum analyzer software, which offered a frequency resolution of 0.5 Hz, and then imported into MATLAB.

**Fig. 6:**
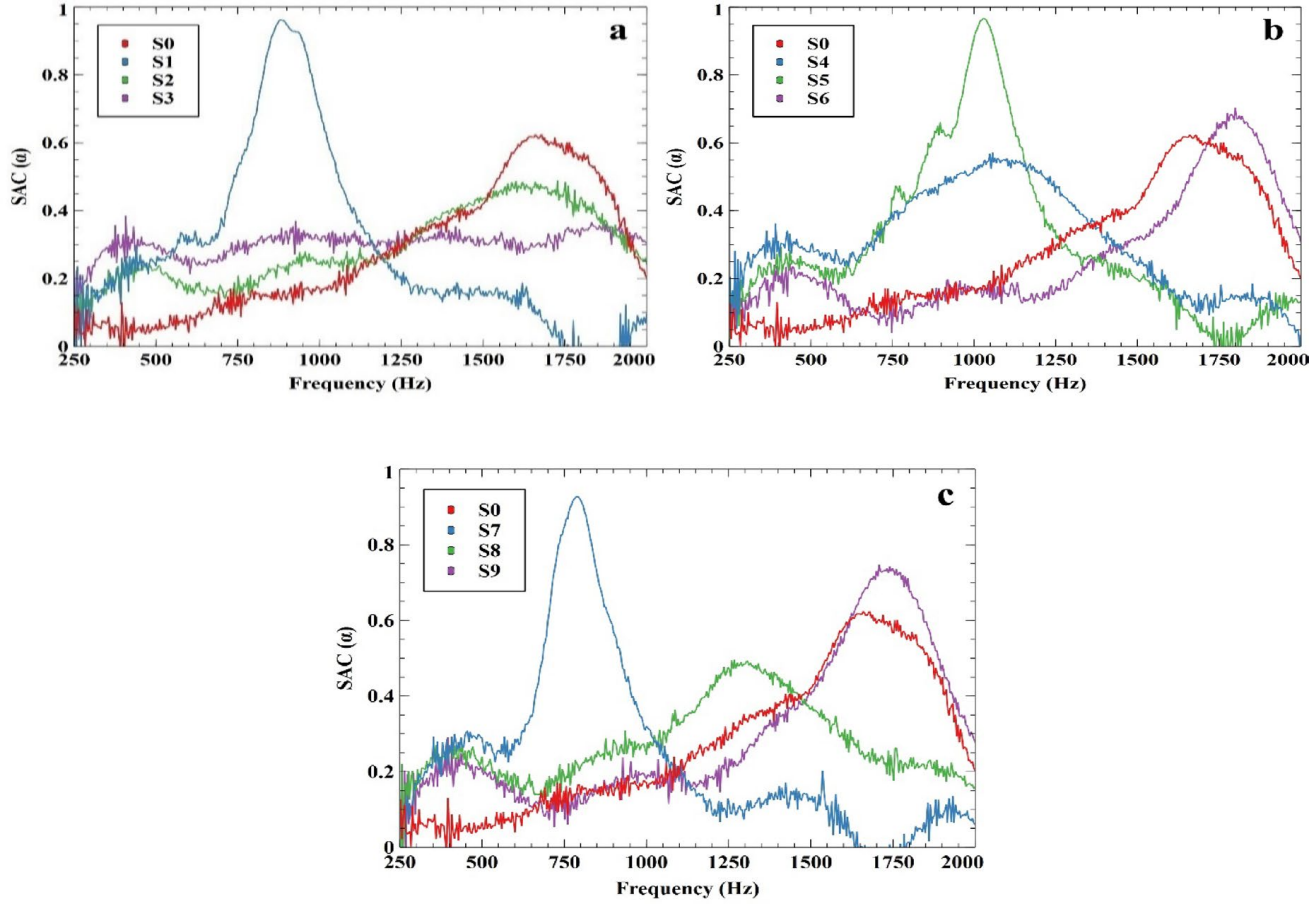
Measurement of SAC with different frequencies for (**a**) Gypsum, (**b**) Vermiculite, and (**c**) RPU powder.

Table 6 lists the SAC values at frequencies of 250 Hz, 500 Hz, 1000 Hz, and 2000 Hz. Figure 6a and c illustrate the similarity in the SAC of samples both with and without fillers. The figures depict sound absorption coefficient (SAC) curves for different samples across a frequency range of 250 to 2000 Hz. In Fig. 6a, samples S0, S1, S2, and S3 are compared. S1 shows the highest SAC peak around 900 Hz, reaching nearly 0.9, indicating excellent sound absorption at this frequency. S0, the control sample, has a relatively low absorption coefficient of around 0.2 across most of the spectrum, while S2 and S3 exhibit moderate absorption with peaks near 0.5 and 0.6, respectively, with S3 performing slightly better at higher frequencies. Figure 6b compares S0, S4, S5, and S6. S4 demonstrates a peak around 900 Hz with an SAC of about 0.85, similar to S1 but slightly higher. S6 shows a pronounced peak at around 1800 Hz with an SAC close to 0.8, indicating superior absorption at higher frequencies, while S5 has moderate absorption with an SAC around 0.4 to 0.5. In Fig. 6c, samples S0, S7, S8, and S9 are analyzed. S7 has a notable peak around 700 Hz with an SAC of approximately 0.9, S9 shows significant absorption at higher frequencies around 1800 Hz with an SAC nearing 0.75, and S8 demonstrates effective absorption over a wide frequency range with peaks around 0.6 to 0.7. Each figure highlights the variations in sound absorption across different samples, both with and without fillers, emphasizing the impact of sample composition on sound absorption performance Table 4.

**Table 4:** Measurement of actual NRC using SAC at different frequencies (Hz).

Samples	SAC at different frequencies (Hz)				Actual NRC
	250	500	1000	2000	
S0	0.017	0.066	0.172	0.203	0.114
S1	0.143	0.237	0.700	0.096	0.294
S2	−0.032	0.226	0.241	0.240	0.169
S3	0.151	0.291	0.316	0.304	0.265
S4	0.150	0.296	0.529	0.033	0.252
S5	0.012	0.229	0.911	0.133	0.321
S6	0.160	0.192	0.171	0.310	0.208
S7	0.091	0.289	0.320	0.062	0.190
S8	0.221	0.240	0.244	0.160	0.216
S9	0.219	0.203	0.180	0.278	0.220
S10	0.219	0.203	0.180	0.278	0.220
S11	0.219	0.203	0.180	0.278	0.220
S12	0.219	0.203	0.180	0.278	0.220
S13	0.219	0.203	0.180	0.278	0.220

The observed NRC values are moderate compared to commercial high-performance acoustic materials but align with expected values for gypsum-based composites. The relatively low absorption is due to the inherent density and structure of gypsum, which limits sound dissipation. However, the inclusion of vermiculite and RPU powder enhances porosity, contributing to increased absorption compared to pure gypsum. Additionally, further optimization, such as modifying pore structure and incorporating additional surface treatments, can improve performance. Future studies may explore alternative processing techniques to enhance porosity and absorption coefficients. Compared to commercial absorbers such as conventional polyurethane foam, which typically exhibit NRC values ranging between 0.4 and 0.6 in mid- to high-frequency ranges^73^ our optimized gypsum-based composites achieved a maximum NRC of 0.3628. This aligns well with mid-level performance while offering enhanced sustainability. Similarly, natural fibre-based materials such as coir have demonstrated SAC values exceeding 0.9 at specific frequencies^74^. Other bio-composites, including jute-reinforced and flax-fibre-based composites, have also reported SACs ranging from 0.6 to 0.85, depending on fiber type and thickness^75^. These comparisons underline the acoustic potential of the proposed composite material, especially in applications where environmental considerations are paramount.

### Optimization using RSM

The credibility of the model was assessed by analysis of variance (ANOVA) and presented in Table 5. The F-values conclude that almost all the results are significant, and the model fits well with the experimental data. It was found that the model predicts the results with more than 90% confidence level.

**Table 5:** Analysis of variance (ANOVA).

Source	DF	Adj SS	Adj MS	F-Value	*P*-Value
Model	5	0.019889	0.003978	42.84	0.000
Linear	2	0.010595	0.005297	57.05	0.000
Vermiculite (Wt.%)	1	0.010553	0.010553	113.65	0.000
Rigid PU Powder (Wt.%)	1	0.000131	0.000131	1.41	0.274
Square	2	0.005186	0.002593	27.93	0.000
Vermiculite (Wt.%)*Vermiculite (Wt.%)	1	0.005177	0.005177	55.75	0.000
Rigid PU Powder (Wt.%)*Rigid PU Powder (Wt.%)	1	0.000916	0.000916	9.86	0.016
2-Way Interaction	1	0.003136	0.003136	33.77	0.001
Vermiculite (Wt.%)*Rigid PU Powder (Wt.%)	1	0.003136	0.003136	33.77	0.001
Error	7	0.000650	0.000093		
Lack-of-Fit	3	0.000650	0.000217	*	*
Pure Error	4	0.000000	0.000000		
Total	12	0.020539			

Table 6 displays both the actual and statistically predicted results for acoustic shielding using the RSM models and regression Eq. 8. The error percentage, calculated with Eq. 7, is also shown in Table 6. The maximum error percentage between the actual and RSM-predicted NRC values relative to the optimal results in Table 6 was under 1.874%. This indicates that regression Eq. 8, based on the RSM analysis, is precise, reflecting a high level of agreement between the actual and predicted results.

**Table 6:** Actual vs. Predicted NRC with error %.

S. No	Vermiculite (Wt.%)	Rigid PU Powder (Wt.%)	Gypsum (Wt.%)	Actual NRC	Predicted NRC	Residual	Error %
S1	5	10	85	0.294	0.302	−0.008	−2.721
S2	15	10	75	0.169	0.162	0.007	3.905
S3	5	30	65	0.265	0.273	−0.008	−2.943
S4	15	30	55	0.252	0.245	0.007	2.698
S5	5	20	75	0.321	0.306	0.015	4.798
S6	15	20	65	0.208	0.222	−0.014	−6.731
S7	10	10	80	0.19	0.189	0.001	0.579
S8	10	30	60	0.216	0.216	0.000	0.139
S9	10	20	70	0.22	0.221	−0.001	−0.227
S10	10	20	70	0.22	0.221	−0.001	−0.227
S11	10	20	70	0.22	0.221	−0.001	−0.227
S12	10	20	70	0.22	0.221	−0.001	−0.227
S13	10	20	70	0.22	0.221	−0.001	−0.227

The optimal Vermiculite, RPU Powder, and Gypsum weight percentages to achieve maximum NRC value are shown in Table 7. Optimum results show that the sample S1 (5.3 Wt.% Vermiculite, 6.5 Wt.% Rigid RPU Powder, and 88.2 Wt.% Gypsum) gives the maximum value of NRC 0.3628. From the experiment, it was found that the sample S1 performs very well at the low frequency range, and it can be used as an effective acoustic shield. Therefore, it can be concluded that the selected weight percentages of these fillers may provide excellent sound insulation properties.

**Table 7:** Optimum results.

Solution	Vermiculite (Wt.%)	Rigid PU Powder (Wt.%)	Actual NRC Fit	Composite Desirability
1	5.3	6.5	0.3628	1

**Fig. 7:**
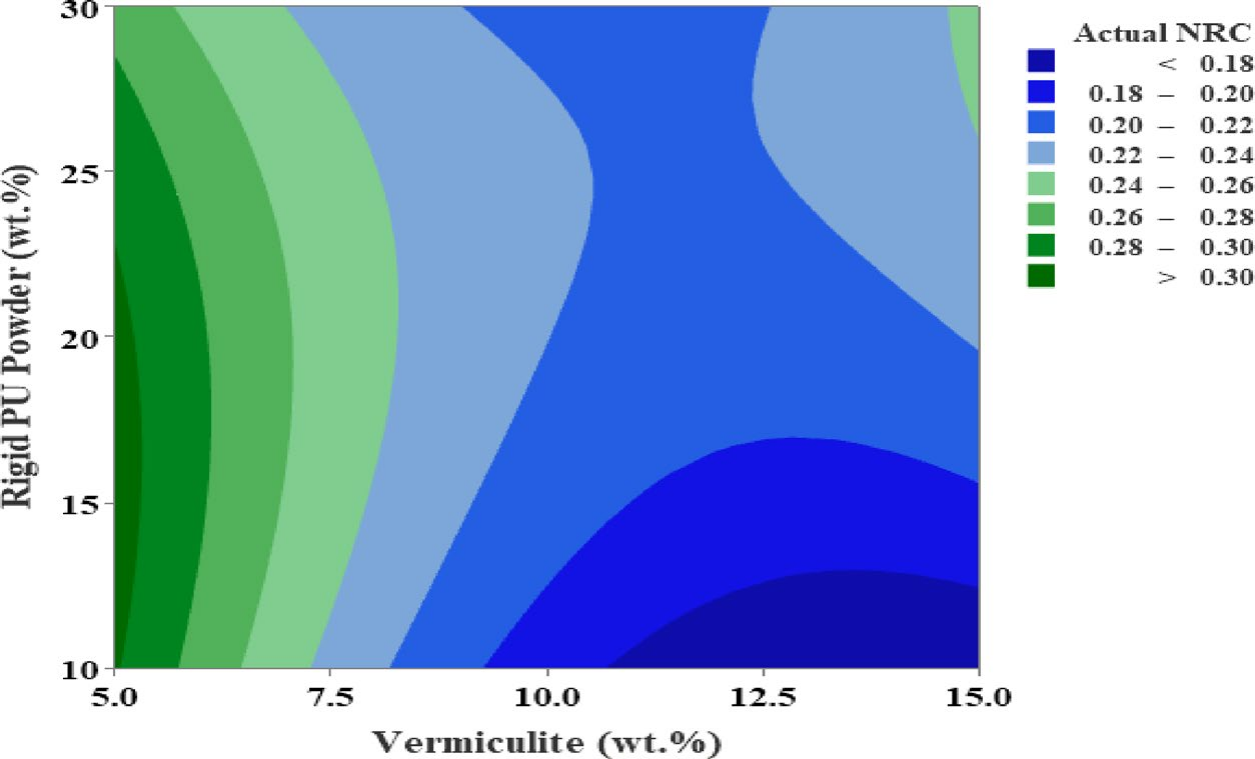
Contour Plot.

**Fig. 8:**
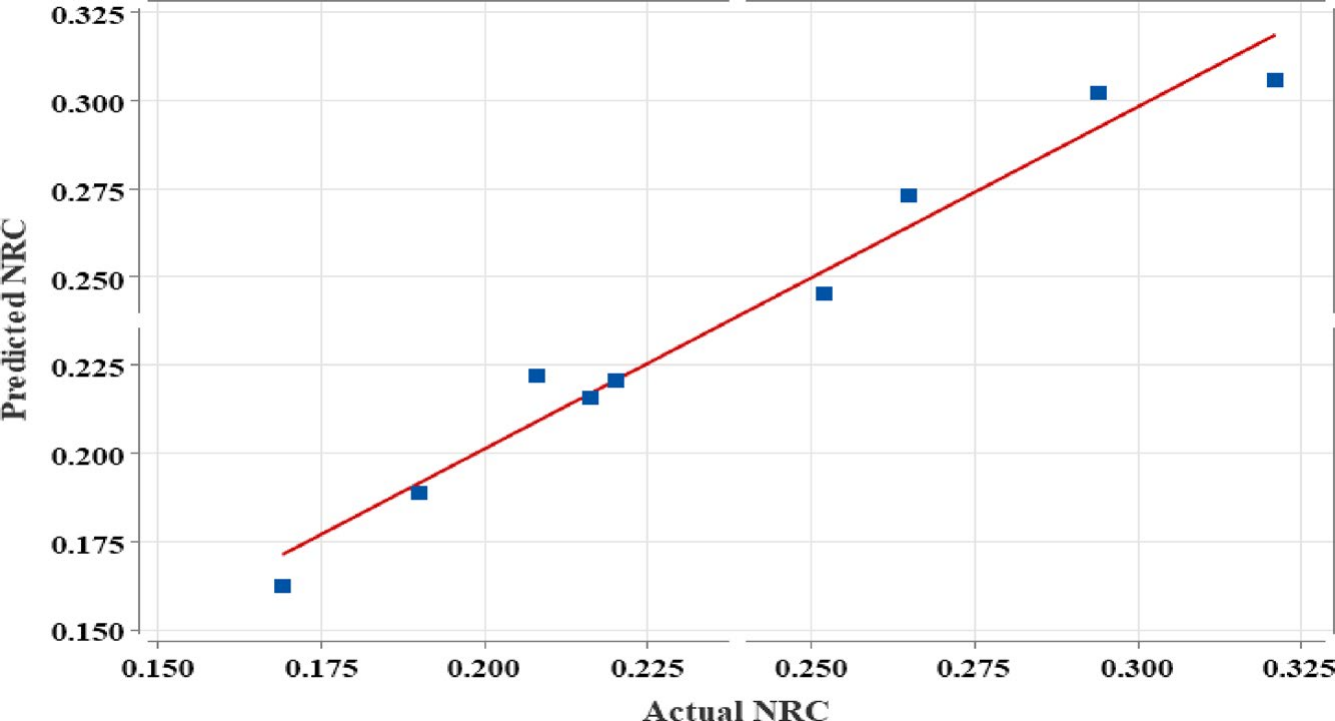
Scatter Plot.

The contour plot in Fig. 7 shows how varying the weight percentages of Vermiculite (5–15%) and Rigid PU powder (10–30%) affect the NRC, with colour gradients from blue (lower NRC, < 0.18) to green (higher NRC, > 0.30). The ideal region for maximum sound absorption (highest NRC) is in the upper left corner, where Vermiculite is low (5-7.5%) and Rigid PU powder is high (25–30%). This combination yields the best Sound absorption efficiency. This scatter plot in Fig. 8 compares the Predicted NRC values (y-axis) against the Actual NRC values (x-axis), with a red line indicating a perfect correlation (y = x). The data points closely align with the red line, suggesting a strong agreement between the predicted and actual values, indicating that the model used for predicting NRC is accurate and reliable. The close clustering around the line implies good predictive performance and minimal error.

**Fig. 9:**
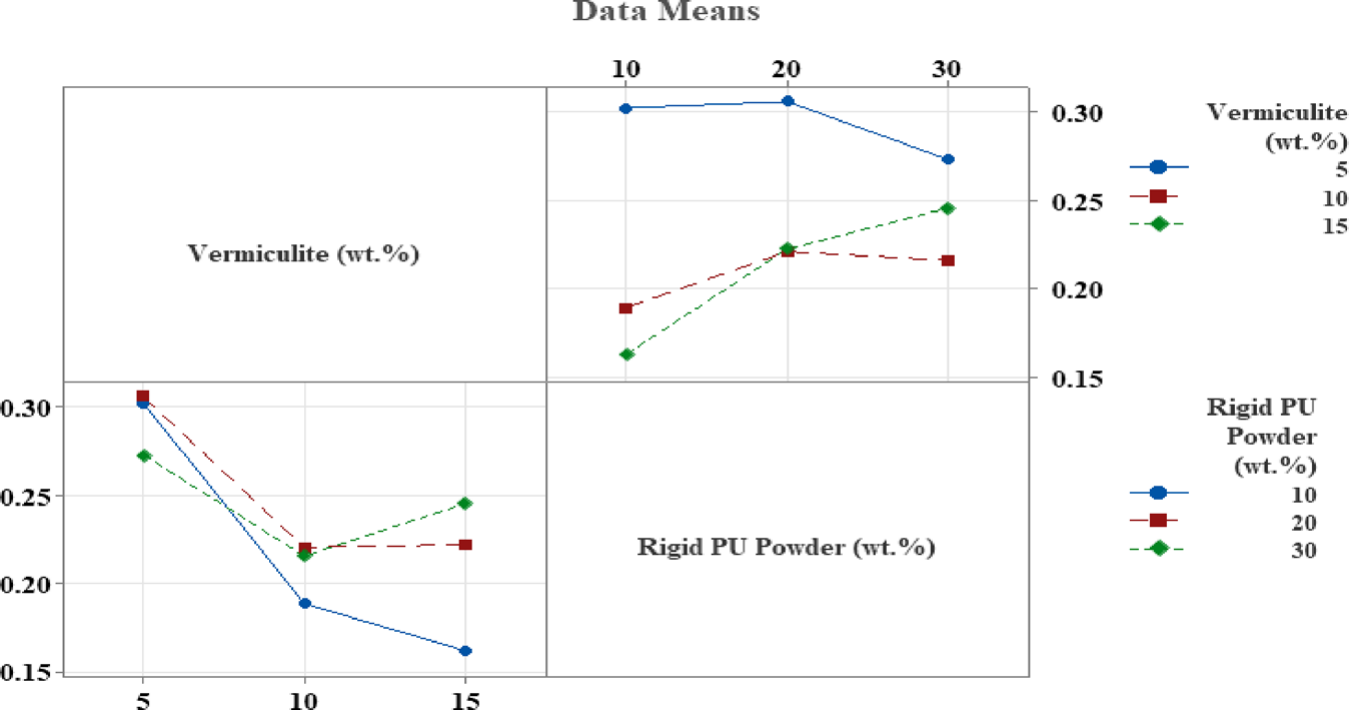
Interaction Plot.

**Fig. 10:**
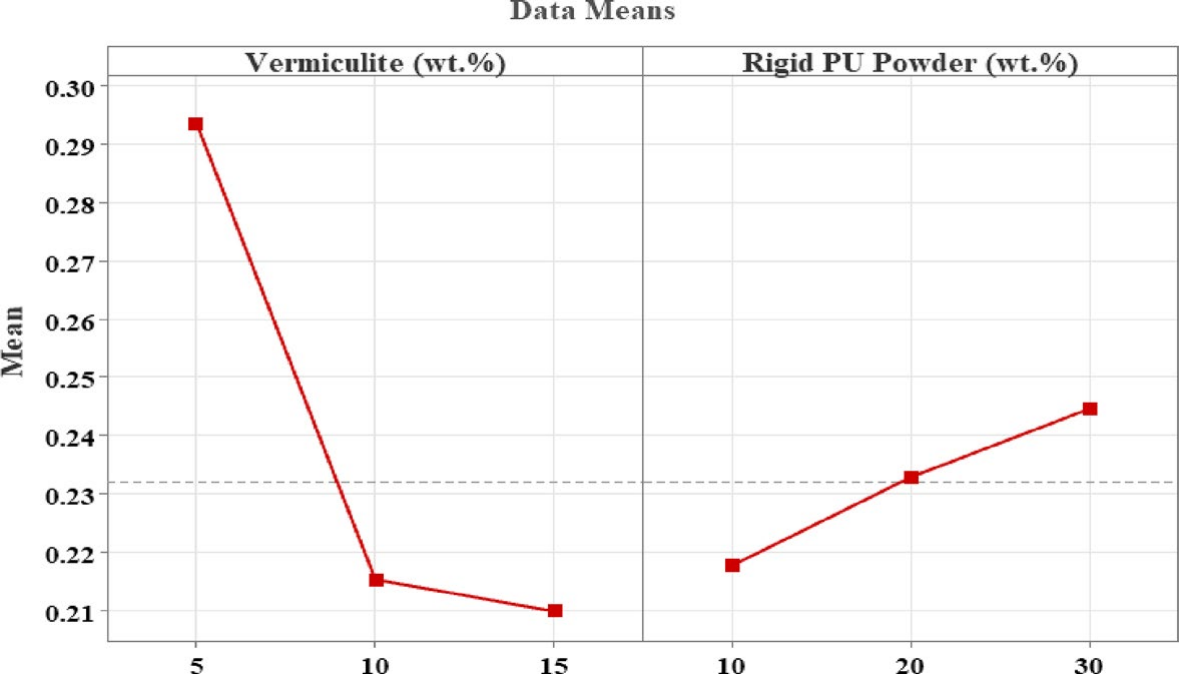
Main Effect Plot.

Figure 9 shows the interaction effects of Vermiculite and Rigid PU powder on the NRC. The top graph indicates how the NRC changes with varying Rigid PU powder percentages (10, 20, 30%) at different fixed levels of Vermiculite (5, 10, 15%). The bottom graph shows how NRC changes with varying Vermiculite percentages (5, 10, 15%) at different fixed levels of Rigid PU powder (10, 20, 30%). Higher NRC values are generally achieved with lower Vermiculite (around 5%) and higher Rigid PU powder (around 30%), as seen in the blue line trends. Figure 10 shows the mean values of a variable for different weight percentages (wt%) of Vermiculite and Rigid PU Powder. For Vermiculite, the mean decreases sharply from 5 to 10% and then slightly decreases further at 15%. For Rigid PU Powder, the mean increases steadily as the wt% increases from 10 to 30%. The dashed line likely represents an overall average or reference value.

**Fig. 11:**
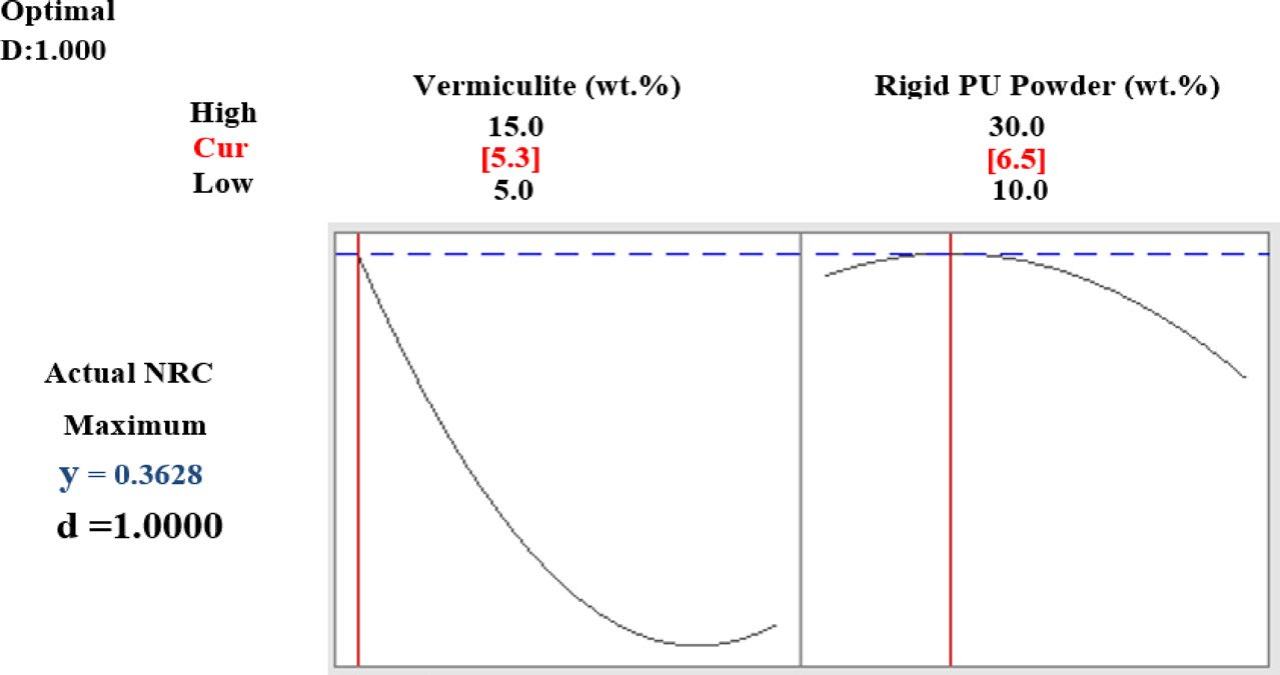
Optimization Curve.

Hence, from the above graphs, we can conclude from Fig. 11 that the mixture contains 5.3 wt% Vermiculite, 6.5 Wt.% RPU Powder, and 88.2 wt% Gypsum yields the maximum NRC value of 0.3628. This obtained maximum NRC value obtained lies in the acceptable significant range, and therefore validates the model.

### Confirmation tests

The optimization results were validated by new test specimens, which were created using the filler weight percentages. Acoustic tests were conducted with these samples, and the results are shown in Table 8. By comparing the predicted and observed values of NRC, a difference of 1.874% was found, which checked the credibility of both the material and the analysis. This, therefore, validates the reliability of the model in predicting NRC at any filler composition and shows future potential for application in improving sound absorption efficiency and related areas.

**Table 8:** Confirmation test on the optimal specimen.

Solution	Vermiculite (Wt.%)	Rigid PU Powder (Wt.%)	Actual NRC Fit	Observed NRC Fit
1	5.3	6.5	0.3628	0.356

### Simulation analysis

 For simulation analyses, both the fundamental properties and those obtained from inverse characterization were utilized. Acoustic simulations of the optimal sample were performed using COMSOL Multiphysics software and the JCA model for acoustic analysis. This model requires values of five parameters: porosity, tortuosity, flow resistivity, viscous characteristic length, and thermal characteristic length as shown in Table 9. These parameters were estimated via inverse characterization using a genetic algorithm. The values for the optimal sample, listed in Table 7, were used as inputs for the COMSOL simulation.

In the modelled system, the dimensions were 5.4 m in height and 4.4 m in width, with the porous layer having a height of 2.8 m. In the COMSOL model, a sound wave strikes the porous absorber at an angle of θ = 0 radians. To handle this incident wave, an air pressure domain is utilized, and a pressure acoustic model is applied to the air domain within the model. Perfectly matched layers are implemented on both sides of the model to absorb all pressure fields. The model was meshed using a physics-controlled mesh with fine-sized triangular elements, ensuring compliance with the six-elements-per-wavelength rule for accurate wave propagation analysis.

**Table 9:** Porosity, tortuosity, flow resistivity, VSL, and TCL by inverse characteristic for the optimum sample.

Porosity	Flow resistivity (Ns/m4)	Tortuosity	VCL (µm)	TCL (µm)
0.850	9971	4.71	239.3	317.8

### Sound pressure level (SPL) simulation

The effect of gypsum composite on sound pressure levels within a room cabin was investigated using COMSOL Multiphysics software. The cabin, measuring 5 m × 4 m × 2.6 m, has a total volume of 52 m³. A monopole point sound source, with a strength of 1 × 10^−5^ m³/s, was positioned at the top of the cabin. The simulation commenced with applying the pressure acoustics frequency domain module to the complete CAD model of the cabin. The gypsum composite’s poro-acoustic properties were defined using the Johnson-Champoux-Allard model, chosen from the software’s dropdown menu. The porous absorber consists of a gypsum composite layer with a thickness of 20 mm, ensuring effective sound absorption within the modelled system. The model was then meshed with a physics-controlled mesh featuring fine triangular elements, and the frequency range from 200 to 2000 Hz was analyzed. This range remains within the practical limits of FEM-based analysis, ensuring reliable results without excessive computational burden.

**Fig. 12:**
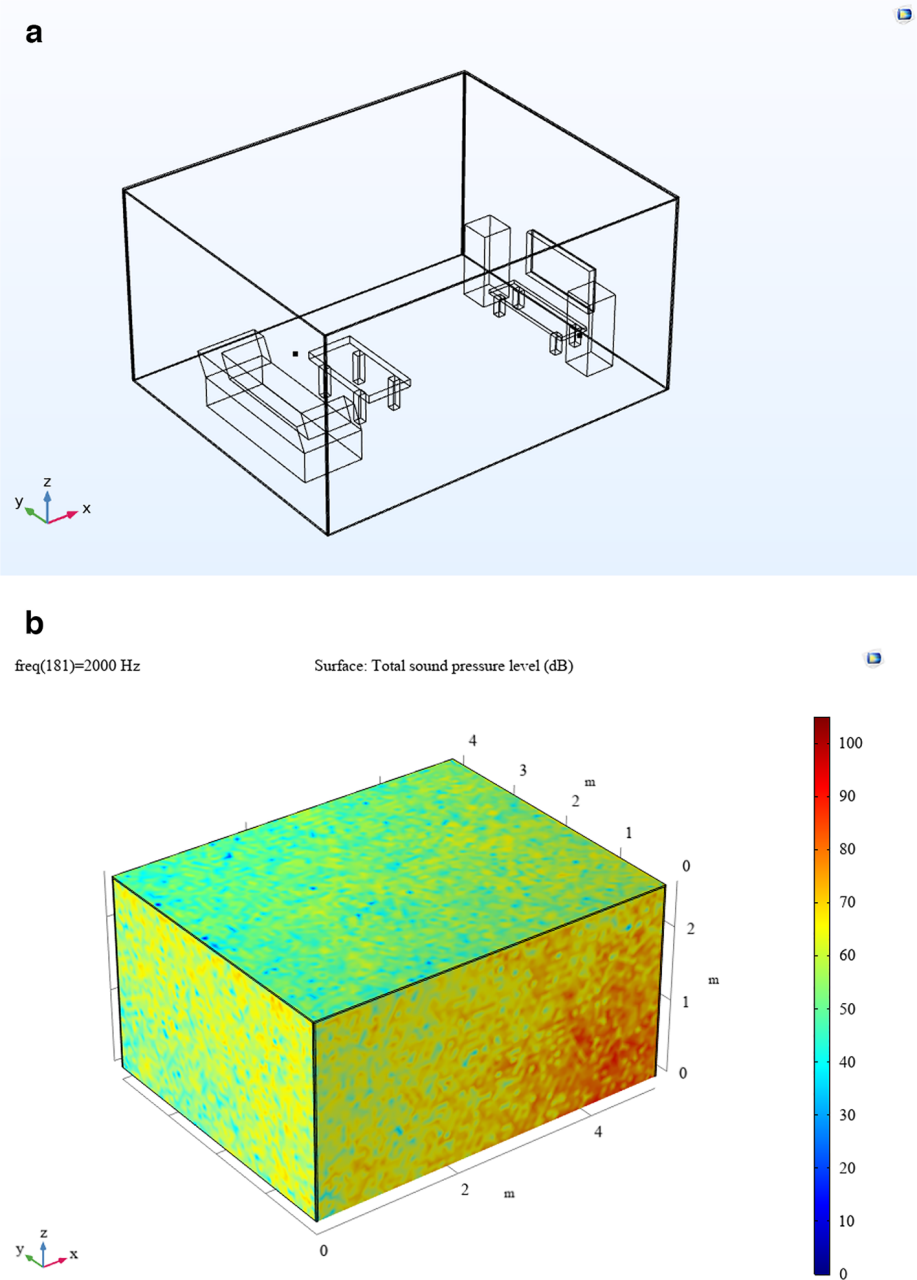
(**a**) Room cabin simulation model. (**b**) Room’s sound pressure level (SPL) simulation.

**Fig. 13:**
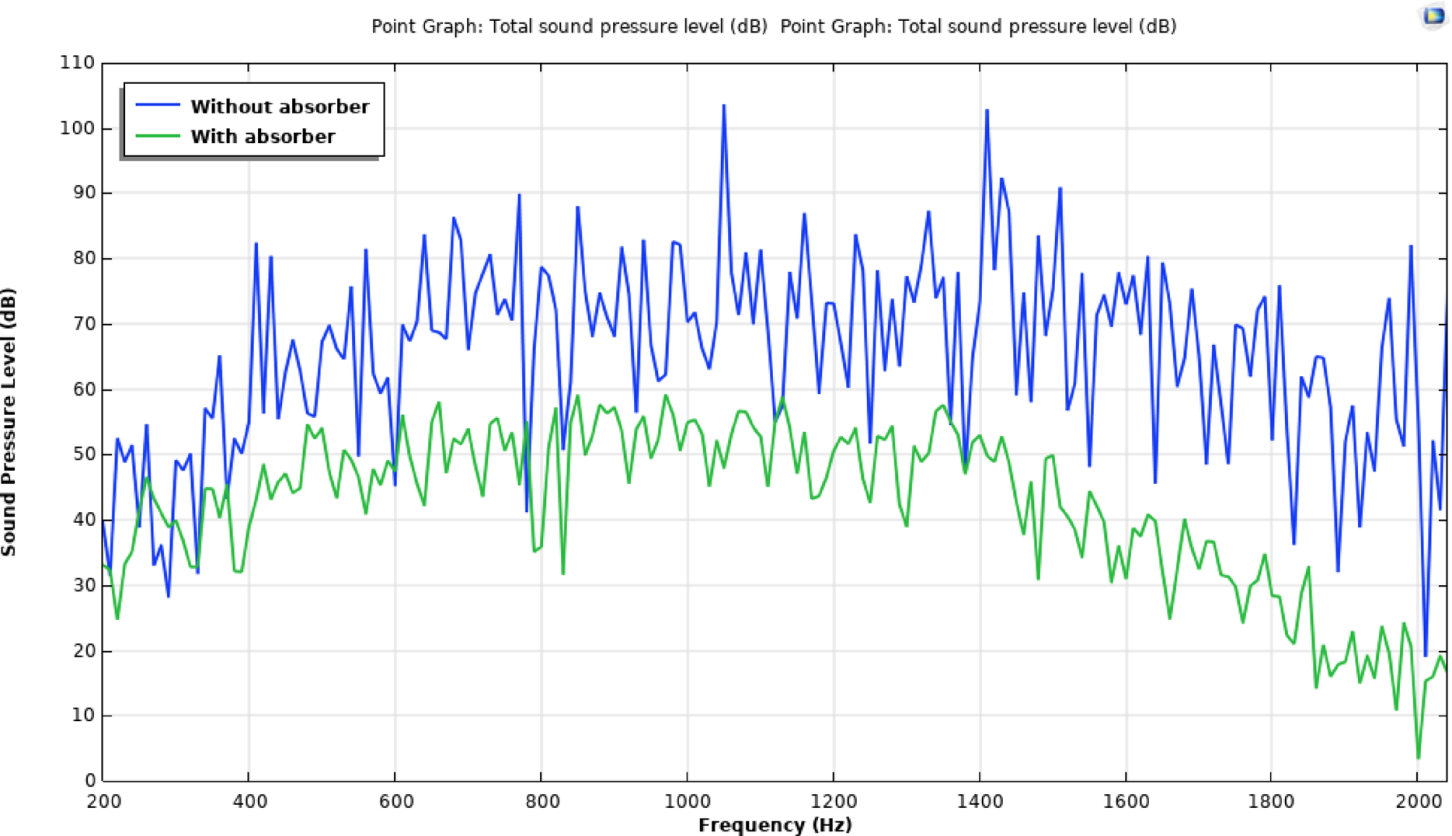
Sound pressure level simulation plot for multilayer configuration.

**Fig. 14:**
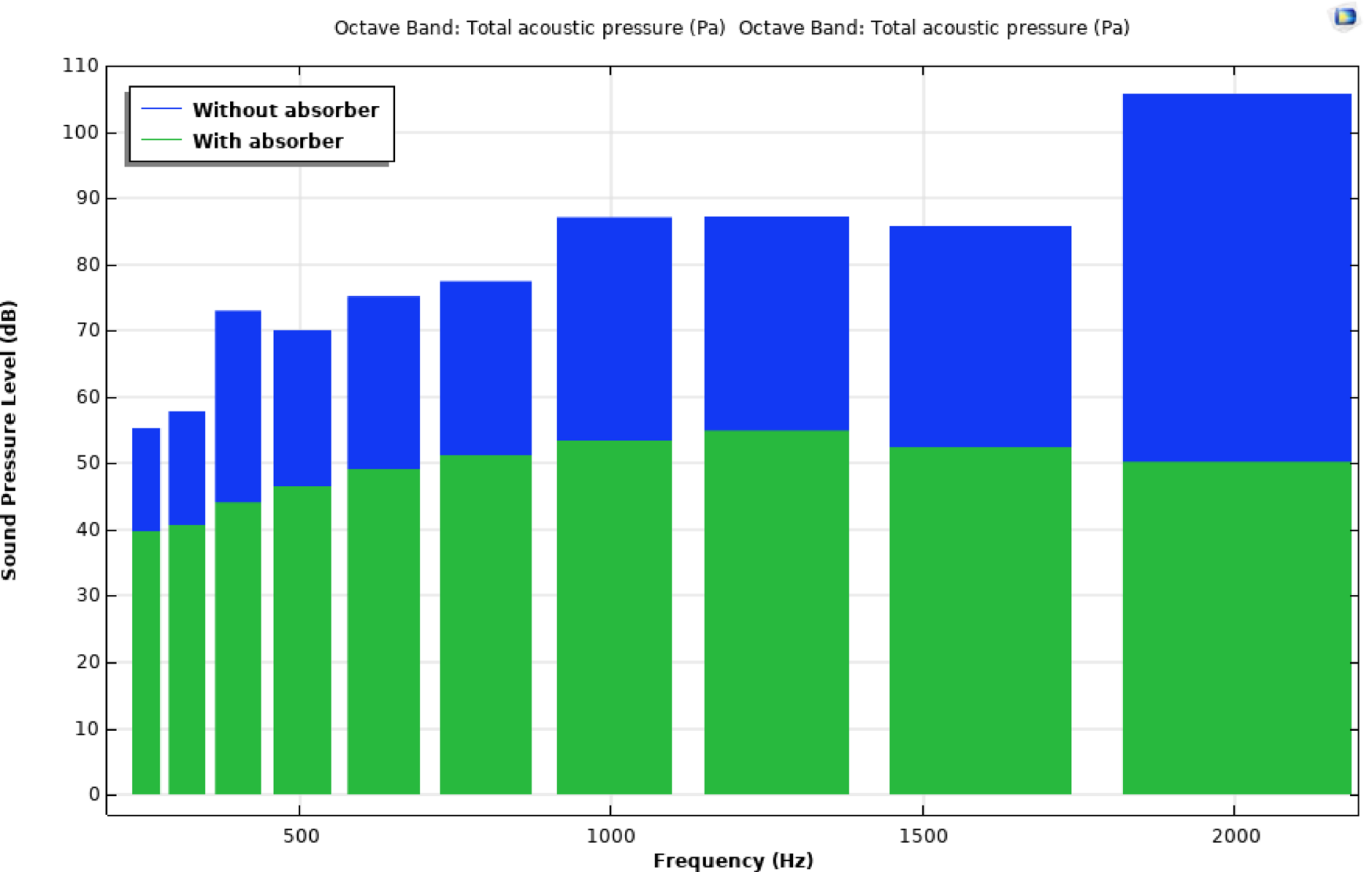
Comparison of SPL plot in one-third octave band.

Figure 12a and b depict the CAD model of the room cabin, where sound pressure level (SPL) measurements were taken at a specific location (P1 = 0.21, 0, 1.28). The simulation outcomes for the cabin, both with and without fillers, are shown in Figs. 12 and 13. Figure 13 illustrates the SPL simulation over the 200–2000 Hz frequency range, while Fig. 14 presents a 1/3rd octave band SPL simulation, created using the octave plot feature in the COMSOL results tab. Figure 14 clearly shows that the SPL inside the cabin was reduced by 17–58 dB with the addition of fillers.

The SPL reduction achieved by our gypsum–vermiculite–RPU composite is comparable to values reported in the literature for commercial absorbers used in architectural settings. For instance, polyurethane-based foam absorbers have been shown to reduce SPL by approximately 30–65 dB depending on frequency and spatial configuration^76^. Although a direct side-by-side comparison was not performed in this study, our results align with the lower-to-mid performance range of these materials, while offering enhanced sustainability.

## Conclusion

A total of 13 samples with different formulations were prepared using the RSM central composite design technique and the blend-press-sinter method. The highest NRC value, 0.3628, was achieved with the composition of 5.3 Wt.% vermiculite, 6.5 Wt.% RPU powder, and 88.2 Wt.% gypsum. The presence of fillers in the gypsum matrix was confirmed through HR-SEM and FTIR analysis. Statistical evaluations using ANOVA identified significant factors affecting the NRC values, which were derived from the regression equation with an error margin of less than 1.874% compared to experimental measurements. The optimized blend of vermiculite, gypsum, and RPU powder offers a sustainable and cost-effective solution for modern construction. Vermiculite and gypsum contribute structural strength and durability, while RPU powder enhances sustainability by utilizing recycled materials. As demand for eco-friendly building materials grows, this composite presents a viable alternative that balances performance, affordability, and environmental impact.

Additionally, SPL simulations conducted using COMSOL software demonstrated an average noise reduction of 17 to 58 dB for the optimal sample compared to an empty cavity. This lightweight and eco-friendly gypsum composite has the potential for applications in sound absorption for automobiles, building walls, industrial machinery, and aerospace insulation. Future research can explore structural modifications, surface treatments, or hybrid material integrations to further enhance its absorption performance and multifunctionality. The comparative analysis—now expanded to include various conventional and natural fiber-based acoustic materials—indicates that bio-composite materials such as coir, jute, and flax demonstrate superior performance in specific frequency bands. Our bio-composite materials exhibit improved sustainability and competitive sound absorption performance compared to many traditional synthetic absorbers. Although the NRC values are slightly below the top range of high-performance commercial synthetic absorbers such as polyurethane foams, our composites demonstrate comparable effectiveness in moderate-frequency ranges, while offering the added advantage of environmental sustainability.

## Data Availability

The datasets generated during and/or analysed during the current study are available from the corresponding author on reasonable request.
